# Neuronal basis of perceptual learning in striate cortex

**DOI:** 10.1038/srep24769

**Published:** 2016-04-20

**Authors:** Zhen Ren, Jiawei Zhou, Zhimo Yao, Zhengchun Wang, Nini Yuan, Guangwei Xu, Xuan Wang, Bing Zhang, Robert F. Hess, Yifeng Zhou

**Affiliations:** 1 CAS Key Laboratory of Brain Function and Disease, and School of Life Sciences, University of Science and Technology of China, Hefei, Anhui, P.R. China; 2 School of Ophthalmology and Optometry and Eye hospital, Wenzhou Medical University, Wenzhou, Zhejiang 325003, P.R. China; 3 McGill Vision Research, Department of Ophthalmology, McGill University, Montreal, Quebec, Canada

## Abstract

It is well known that, in humans, contrast sensitivity training at high spatial frequency (SF) not only leads to contrast sensitivity improvement, but also results in an improvement in visual acuity as assessed with gratings (direct effect) or letters (transfer effect). However, the underlying neural mechanisms of this high spatial frequency training improvement remain to be elucidated. In the present study, we examined four properties of neurons in primary visual cortex (area 17) of adult cats that exhibited significantly improved acuity after contrast sensitivity training with a high spatial frequency grating and those of untrained control cats. We found no difference in neuronal contrast sensitivity or tuning width (Width) between the trained and untrained cats. However, the trained cats showed a displacement of the cells’ optimal spatial frequency (OSF) to higher spatial frequencies as well as a larger neuronal signal-to-noise ratio (SNR). Furthermore, both the neuronal differences in OSF and SNR were significantly correlated with the improvement of acuity measured behaviorally. These results suggest that striate neurons might mediate the perceptual learning-induced improvement for high spatial frequency stimuli by an alteration in their spatial frequency representation and by an increased SNR.

Extensive training improves the performance on the trained feature, a phenomena which is known as perceptual learning. It has been well accepted that this occurs through the enhancement of the modulation in neuronal tuning to stimulus components that are relevant to the task^1^. Moreover, the learning effects transfer to stimuli other than the trained stimulus in some cases. Of the particular interest is the report that contrast sensitivity training at a high spatial frequency (SF) results not only in improvements of contrast sensitivity at the trained frequency, but also improvements in acuity for both gratings and letters^2^,^3^,^4^. While it is known that improved neuronal contrast sensitivity offers an explanation for the improvements in behavioral contrast sensitivity induced by perceptual learning at low spatial frequencies^5^ where acuity is unaffected, little is known of the neural basis of the perceptual learning effects for high spatial frequencies where acuity is also improved. Here we consider four possible neural explanations for the direct and transferred improvements found following perceptual learning at high spatial frequencies: 1) increase of the average contrast sensitivity of neurons tuned to high spatial frequencies^6^; 2) increase in the number of neurons responding to high spatial frequencies^7^,^8^,^9^, which shows as increased optimal spatial frequency (OSF) (Fig. 1a); 3) a broadening of the spatial frequency tuning response of individual neurons, which increases the response at high spatial frequencies (Fig. 1a), and 4) improved neuronal signal/noise ratio (SNR; Fig. 1a), in which maximal responses (Rmax) for high spatial frequency stimuli increased and/or spontaneous activity (M) decreased.

To answer these questions, we trained four adult cats to improve their contrast sensitivity at a high spatial frequency (i.e., 1 c/d) using an orientation identification task. The training protocol was similar to the one originally introduced and validated by Mitchell *et al.*^10^,^11^,^12^. Before and after the training stage, we measured each cat’s grating acuity for the two eyes separately (Fig. 1b). Additionally, electrophysiological recording was conducted after the training stage in three trained cats and four untrained control cats. We found that training significantly improved the visual acuity of trained cats similar to what is already known in humans^2^,^3^. Furthermore, for trained cats as opposed to untrained cats, the striate neurons’ spatial frequency tuning properties was shifted to higher optimum spatial frequencies and better SNR. Moreover, the psychophysical changes and physiological differences were correlated suggesting that the acuity enhancement obtained by training at high spatial frequencies is the result of an alteration of both the spatial frequency representation and SNR in striate neurons.

## Results

### Training effect at the behavioral level

Before the start of the experiment, cats were familiarized with the visual tasks (conditioning stage). In this conditioning stage, four cats learnt to identify the orientation of a high-contrast sinusoidal grating at 0.3 or 0.4 cycle/degree (c/d) within 8 weeks to a performance accuracy at or higher than 90%. Then, they were monocularly tested at a range of spatial frequencies set to 90% contrast to obtain the behavioral spatial frequency response curves (Figure S1 in Supplementary). Visual acuities were then derived by calculating the spatial frequency corresponding to 75% accuracy. Statistical analysis showed that, before the training, the visual acuity was not significantly different between the two eyes of trained cats (Paired t test, t (3) = 2.75, *p* = 0.071).

After these initial baseline measurements, cats were trained monocularly at 1.0 c/d using the same task as the conditioning stage for about forty days. A staircase procedure was used to track the threshold contrast of the grating for each cat over the entire training course. As expected, contrast sensitivity at the trained spatial frequency systematically increased for all the cats, by 15.34 ± 6.45 dB (Mean ± SD) on average (Fig. 2a). Training also increased the grating acuity of trained eyes from 1.08 ± 0.16 c/d to 1.46 ± 0.21 c/d (Mean ± SD; Paired t test, t (3) = 3.453, *p* = 0.040, Fig. 2b), and that of untrained eyes from 1.15 ± 0.17 c/d to 1.34 ± 0.17 c/d (Mean ± SD; Paired t test, t (3) = 3.326, *p* = 0.044, Fig. 2c). Moreover, the increase of visual acuity was not significantly different between eyes (Two way ANOVA, F (1, 12) = 0.987, *p* = 0.34).

### Training effect on V1 neurons

In total, we recorded from 190 cells from three trained cats and 183 cells from four untrained control cats. The recording depths were comparable in two groups (X^2^(9) = 14.20, *p* = 0.115). Neurons within 8° of eccentricity were recorded and the eccentricity distribution of cells was matched between the two groups (Individual samples t-test, t (371) = 0.426, *p* = 0.67). The distribution of preferred orientation was also not significantly different between the two groups (X^2^(18) = 23.12, *p* = 0.186). Since the increase of visual acuity was not significantly different between eyes and the neuronal properties that were recorded from trained eyes were similar to that from untrained eyes in trained cats, we combined the neural recordings obtained through the two eyes in the trained cats. In addition, the training-related effects were quite similar for simple and complex cells^13^,^14^ (Figure S2 in Supplementary), so data was collapsed across cell type.

### Effects of Training on Contrast Sensitivity of striate Neurons

In Fig. 3a, neurons’ contrast sensitivities are plotted as a function of spatial frequency for the two groups. Neurons were clustered into seven groups based on their optimum spatial frequency. Contrast sensitivity of individual neurons was defined as the inverse of each neuron’s threshold contrast sensitivity, which was obtained by receiver operating characteristic (ROC) analysis^15^ (See Supplemental method). Surprisingly, we did not find any significant differences of threshold contrast sensitivity between the trained and untrained groups (F (1,359) = 0.011, *p* = 0.917, Fig. 3a). Furthermore, we found no significant interaction between training and stimulus spatial frequency for neuronal threshold contrast sensitivity (F (6,359) = 1.601, *p* = 0.146), which means that the shape of the contrast sensitivity function was invariant between the two groups. These results suggest that the improved visual acuity we observed is not likely to be accounted for by a change in neuronal contrast sensitivity of individual striate neurons.

### Effects of Training on SF Tuning Characteristics of Single Neurons

Since the neuronal spatial frequency tuning characteristics may affect the visual acuity^16^,^17^, we derived the optimal spatial frequency (OSF), tuning bandwidth and SNR for each neuron from its spatial frequency response function (See Methods) and compared these between the trained and untrained cats. Our results indicated that the trained cats showed a significant alternation in the optimal spatial frequency (OSF) distributions of striate neurons, with an obviously shift in the location of the peak response towards higher frequencies compared to control cats (Fig. 3b, Mann-Whitney U test, *p* < 0.0001). This means that more cells responded to high spatial frequency stimulation in trained cats. In particular, the proportion of cells for which the preferred spatial frequency was around that of the trained spatial frequency (0.9–1.1 c/d) increased in the trained cats (15.263%) compare to the untrained cats (6.557%). The average optimum spatial frequency (OSF) was 0.70 ± 0.03 c/d and 0.55 ± 0.02 c/d (Mean ± SE) in the trained cats and untrained cats (t (371) = 4.54, *p* < 0.0001), respectively (Fig. 3b).

For the comparison of tuning bandwidth and SNR between the trained and untrained groups, we grouped neurons into one of three classes based on their optimum spatial frequencies: low spatial frequency neurons with optimum spatial frequencies smaller than 0.45 c/d, medium spatial frequency neurons with optimum spatial frequencies between 0.46 to 0.85 c/d and high spatial frequency neurons of which optimum spatial frequencies higher than 0.85 c/d. The results that are plotted in Fig. 3c,d show that training had no significant effects on spatial frequency tuning bandwidth (F (1,367) = 1.732, *p* = 0.189, Fig. 3c), but did improve the SNR of striate neurons (F (1,367) = 9.904, *p* = 0.002, Fig. 3d). Additionally, there were no significant interaction between training effect and spatial frequency for tuning bandwidth (F (2,367) = 0.621, *p* = 0.538) and SNR (F (2,367) = 0.282, *p* = 0.755).

We further examined whether the training effects at the single cell level were themselves eccentricity-dependent. We found that the above mentioned training effects occurred in both the central (0–4 deg) and peripheral eccentricities (4–8 deg): OSF (central area: t (156) = 3.693, *p* < 0.001; peripheral area: t (213) = 2.747, *p* = 0.007) and SNR (central area: t (156) = 2.531, *p* = 0.012; peripheral area: t (213) = 2.368, *p* = 0.019) in the trained cats were significantly higher than those in control cats. Further analysis showed that the interaction between training and eccentricity was not significant (F (1,369) = 1.227, *p* = 0.269 for OSF and F (1,369) = 0.259, *p* = 0.611 for SNR).

### Were the learning effects limited to the orientation of the target

So far we have demonstrated that the optimum spatial frequency and SNR of striate neurons were increased in trained cats, one critical question is whether these differences we observed were limited to neurons whose orientation preference corresponded to that of the target orientation. In the analysis to date we have assumed that orientation is unimportant because neuronal properties have been averaged across neurons with different oriental preferences. To examine this, we divided the neurons into two groups: neurons with optimal orientation near (±20°, NN) the target orientation and those away from the target orientations (NA). Interestingly, we did not find any significant difference in post-training neuronal properties between NN and NA in the trained cats: Threshold contrast sensitivity (t (188) = 0.808, *p* = 0.420), optimum spatial frequency (t (188) = 1.49, *p* = 0.138), tuning bandwidth (t (188) = 0.679, *p* = 0.498) and SNR (t (188) = 1.063, *p* = 0.289). The two-way ANOVA analysis also showed that there were no significant interaction between training and orientation on threshold contrast sensitivity (F (1,369) = 0.894, *p* = 0.345), optimum spatial frequency (F (1,369) = 2.872, *p* = 0.091), tuning bandwidth (F (1,369) = 1.153, *p* = 0.284) and SNR (F (1,369) = 0.281, *p* = 0.596) for the comparison of trained and untrained cats (Fig. 4). These results indicated that the neuronal differences in the training group were not orientation specific.

### Correlation between the perceptual changes and neuronal correlates

Figure 5 shows the correlation between the perceptual and ‘net’ neuronal changes in the three trained cats from which we recorded. The average neuronal responses of untrained cats were taken as baseline to calculate the ‘net’ improvement. The increase of neuronal optimal spatial frequency and the improvement of perceptual visual acuity from each trained and untrained eye in the three trained cats were highly correlated (Fig. 5a, *r* = 0.98, *p* < 0.0001). The enhancement of neuronal SNR and the improvement of visual acuity were also significantly correlated (Fig. 5b, *r* = 0.78, *p* = 0.020). Further analysis shows that it is the decrease of M (Fig. 5c, *r* = 0.72, *p* = 0.034) rather than the increase of Rmax (*r* = 0.04, *p* = 0.734) that drove the significant correlation with the visual acuity improvement, which in turn means that the decrease of neuronal spontaneous activity may contribute to the behavioral improvement.

## Discussion

Through behavioral assessment and subsequent physiological recording in trained and untrained cats, we have been able to show that contrast sensitivity training at a high spatial frequency significantly improves the visual acuity of cats and that these changes are correlated with ‘net’ changes in the properties of striate neurons. Specifically, at the cellular level, trained cats showed a significant shift of the optimum spatial frequency distribution of striate neurons toward higher spatial frequencies and also a larger SNR of striate neurons compared to control cats. We suggest that the altered spatial frequency representation and the augmented SNR in striate neurons might underlie the improvement of visual acuity during perceptual learning through contrast sensitivity training at high spatial frequencies.

In behavior experiments, the head position of cats may cause a variation of observation distance especially when this distance is short (57 cm). This would lead to a fluctuation of training/testing SF. This fluctuation on training/testing SF would have been very small because a change in head position greater than, for example, 5 cm, was rarely observed, thus the SF of our targets was within 10% of the plotted value. Cats were not observed to take up different head positions after training.

Another issue that is worth noting is that to compare the current study with a previous study^5^ on perceptual learning in cats, the mean luminance in our study was 19 cd/m^2^. This in itself is unlikely to bear on the training results shown here as previous training improvements have been shown for stimuli modulated on a large range of luminance^3^,^18^,^19^. However, it would contribute to the reduced acuities^20^ we obtain in ours cats compared with previous studies^11^,^12^.

For cellular measures, all cats were anesthetized and paralyzed. While this is likely to affect accommodation and pupil size, however, since trained and untrained cats were subjected to the same protocols these cannot provide a suitable explanation for the training induced effects.

The averaged OSF value of A17 neurons within 8 degrees was 0.55 ± 0.02 c/d (Mean ± SE) in our untrained cats. This result is comparable with previous investigations within similar eccentricities^5^,^21^,^22^, which ranged from 0.5 to 0.6 c/d. For example, in Hua *et al.*’s study^5^, the average neuronal OSF of A17 in normal cats was about 0.51 c/d and most neurons were around 0.4 c/d. Using optical imaging, Issa *et al.*^22^ reported that the distribution of optimal SFs of A17 within 8.5° in adult cats ranged from 0.1 to 1.84 c/d with a median value of 0.53 c/d. Also, Ribot *et al.*^21^ mapped spatial frequency of A17 within 8.5° in adult cats using optical imaging and found that the median value of OSF was 0.56 cpd ± 0.34 octave median absolute deviation. However, compared to the study of Movshon *et al.*^23^, where the OSF of neurons within 8 degrees in A17 of cats spanned a range from 0.25 to more than 2 c/d with a mean value of about 0.68–0.7 c/d, our OSF distribution of normal cats seemed biased towards low SFs. However, since we derived our OSF values from spatial frequency response curves that were fitted with logGaussian function while they extracted neuronal OSF from contrast sensitivity data by eye, there may be a methodically explanation.

Our results suggest that the primary visual cortex plays an important role in visual perceptual learning. There are also studies in monkeys^24^ and in human^25^ showing that visual perceptual learning induces corresponding changes in the primary visual cortex. For example, Schouops *et al.*^24^ found that training on a fine orientation discrimination task could lead to sharpening of orientation tuning of V1 neurons. While our task involved contrast training at a high SF, we also found neuronal differences that are relevant to the processing of high SF stimuli. Using human functional magnetic resonance imaging (fMRI), Furmanski *et al.*^25^ showed that training in contrast detection elevated V1 activity and suggested that training might increase the number of neurons responding to the trained stimulus or increase the response gain in human cortex. In the current study, we show that the neurons responding to high spatial frequencies increased and the neuronal SNR improved in the trained cats compared to those in control cats , complimenting Furmanski *et al.*^25^’s observation.

Interestingly, our results are different from that of Hua *et al.*’s study^5^, in which the differences of neuronal contrast sensitivity in primary visual cortex were identified as the possible cause of the behavioral improvement. In this previous study, perceptual learning was undertaken at a low-mid spatial frequency, unlike the high spatial frequency used in the current study. It is well known in humans^2^ and confirmed in cats^5^ that training at these lower spatial frequencies does not produce changes in acuity, unlike training with a high spatial frequency stimulus. When training is undertaken at a high spatial frequency, we did not find that differences in neuronal contrast sensitivity offered a suitable explanation for the observed behavioral improvements, rather it was the differences of optimum spatial frequency of cells as well as their SNR that best explained the acuity improvements documented behaviorally. On the basis of this it would seem that the neural improvements from perceptual learning might depend on whether a high or low spatial frequency is used for training, a conclusion consistent with the psychophysical differences already documented in humans^4^.

An expansion of the cortical representation has also been suggested as a mechanism of perceptual learning in other sensory domains^7^,^8^,^9^,^26^,^27^. Our study is consistent with this in showing a significantly increased representation of high spatial frequencies achieved by a systematic shift in the optimum spatial frequency of neurons towards higher spatial frequencies. Although large-scale spatial reorganization of the cortical network has been previously reported for the auditory^7^,^26^ and somatosensory cortex^8^,^9^, this is the first report of a comparable change for the visual cortex.

In addition, many studies reported that either no or minimal changes in primate V1 following learning^24^,^28^, the neural changes, however, occurred mainly in V4^29^,^30^,^31^,^32^, MT^33^ or some higher areas^34^,^35^,^36^,^37^. For example, using orientation discrimination task, Adab and Vogels^29^, Raiguel *et al.*^30^ and Yang *et al.*^31^ reported significant neuronal changes in V4. Zohary *et al.*^33^ found that the directionally selective sensitivity of MT neurons increased after training in tasks that involved discrimination of motion direction. Sigala and Logothetis^36^ showed that training monkeys to categorize visual stimuli based on different features led to an enhanced neuronal representation of the diagnostic features in the responses of IT neurons. There are three critical differences between our study and this previous literature: (1) Different species: we used nonprimates (cats) whereas neurophysiological studies of visual perceptual learning in the literature all used primate subjects. Cats have a poorer visual system than primates, thus they might exhibit more training-induced plasticity in early visual areas^5^,^38^. (2) Different tasks: many studies have shown that training-induced neural plasticities are task-dependent^1^,^39^,^40^. In contrast to previous studies that generally used orientation discrimination task^29^,^30^,^31^, or more complicated tasks such as venier discrimination and contour detection^41^,^42^, the current study used a more basic detection task that relies on contrast and orientation information, yielding a result quite different from those cited above. (3) Different animal states: previous studies were performed in awake-behaving monkeys, whereas the current data are from anesthetized and paralyzed cats. Compared to studies on anesthetized animals, recordings from early visual cortex of awake animals would be expected to have more top-down influences from higher visual cortical areas^41^,^43^,^44^.

The finding that training effects did not show obvious orientation specificity is not unique and is consistent with many psychophysical studies^19^,^45^,^46^. There is much psychophysical support for the notion that visual filters are tuned for spatial frequency but pool information across orientation^47^,^48^,^49^,^50^. For example, Georgeson and Meese^48^ suggested that the summation across orientations occurs at earlier site than the summation across different spatial frequencies. It is quite possible that our training paradigm induced neuronal changes mainly at this earlier site, and that this is why we did not show any clear orientation dependency.

To conclude, our study showed that training in contrast sensitivity at high spatial frequencies not only improved the contrast sensitivity at the trained spatial frequency but also increased the visual acuity of the trained cats, similar to that already known from human psychophysics. Furthermore, the optimum spatial frequency and SNR of striate neurons were increased in the trained cats. The increase of optimum spatial frequency and SNR of striate neurons were significantly correlated with the visual acuity improvements, suggesting a possible neural substrate.

## Methods

### Animals

Eight adult cats (age: 1–2 years old; body weight: 2.3–3 kg) were studied in this study: four cats were involved in visual training tasks and three of them underwent subsequent electrophysiological single unit recording. The other four untrained cats were subjected to extracellular single unit recording as the control group. All cats were examined ophthalmoscopically to confirm that they had no optical or retinal problems. All surgical and experimental procedures were in accordance with the National Institute of Health guidelines for the humane use and care of animals and were approved by the Institutional Animal Care and Use Committee of University of Science and Technology of China.

### Psychophysical Procedures

**Figure 1:**
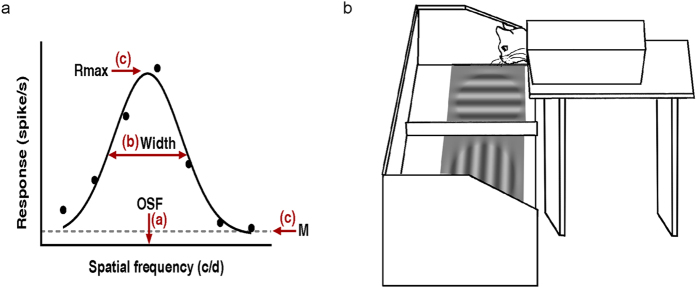
Possible changes in neuronal SF tuning after training and schematic diagram of apparatus for training cats. (**a**) Possible mechanisms in neuronal SF tuning underlying the visual acuity improvement. (1) Increase of optimal spatial frequency (OSF). Learning may increase the number of cells in the cortical population that prefer the trained SF, which means that the OSF of neurons in the trained cats would shift to spatial frequencies matching the trained spatial frequency. (2) Increase in tuning width (Width). Training may increase Width so that the response to the high SF increases. (3) Improvement of signal-to-noise ratio (SNR). SNR is defined as Rmax (fitted maximal visually evoked response)/M (measured spontaneous activity). Learning may lead to an increase of Rmax and/or a decrease of M, which result in an increase in SNR. (**b**) Cats were trained monocularly to walk through a box and jumped onto the glass above a monitor on which two orthogonal stimuli were displayed. Jumps to the vertical one were rewarded with food and petting, whereas the horizontal one resulted in denial of the rewards and immediate next trial. In the training stage, frequency of the grating was relatively high and remained unchanged for each cat. A staircase procedure was used to track the threshold contrast of the grating for each cat over the entire training course. Visual acuities (grating acuities) of the two eyes were measured before and after the training stage.

**Figure 2:**
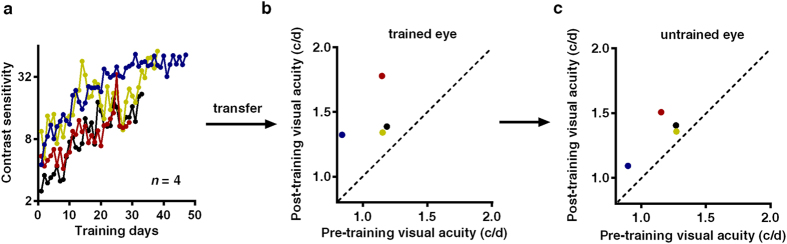
Training not only increased contrast sensitivity at the trained SF, but also improved the cat’s visual acuity. (**a**) Learning curves of the four trained cats. Each color represents one cat. (**b,c**) Post- versus pre-training visual acuity for trained eyes and untrained eyes in trained cats, respectively. Each dot represents one eye. The points are above the dashed line (slope = 1), indicating both the visual acuity for trained and untrained eyes in trained cats improved after training.

**Figure 3:**
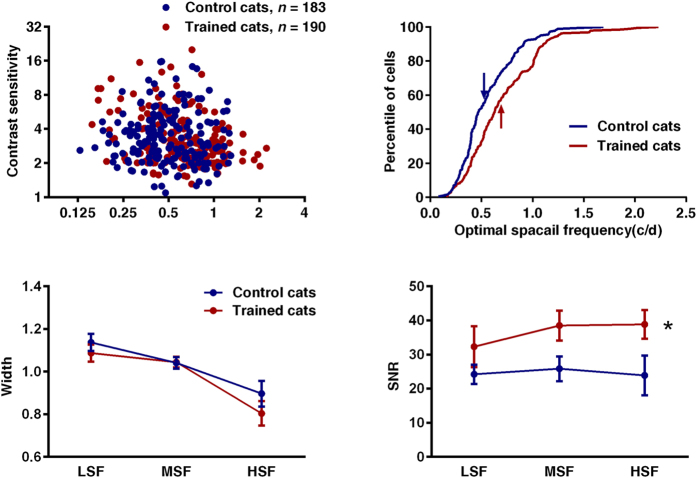
Training effect on striate neurons. Blue and red colored plots represent control and trained cats, respectively. LSF, MSF and HSF indicate neurons with low spatial frequency (<0.45 c/d), medium spatial frequency (0.45–0.85 c/d) and high spatial frequency (>0.85 c/d), respectively. (**a**) There was no significant difference between the threshold contrast sensitivity (TC) of striate neurons from control and trained cats. (**b**) There was significant difference between the OSF distribution for striate neurons from control and trained cats. The arrows indicated the mean of OSF values (0.55 ± 0.02 c/d for control cats, and 0.70 ± 0.03 c/d for trained cats) (mean ± SE). (**c**) Neither significant training effect nor interactions between training and SF were found in Width between trained and control cats. Error bars indicate SEM. (**d**) Significant difference was showed in SNR between control and trained cats (Two way ANOVA, F (1,367) = 9.904, *p* = 0.002). ‘*’indicates *p* < 0.05.

**Figure 4:**
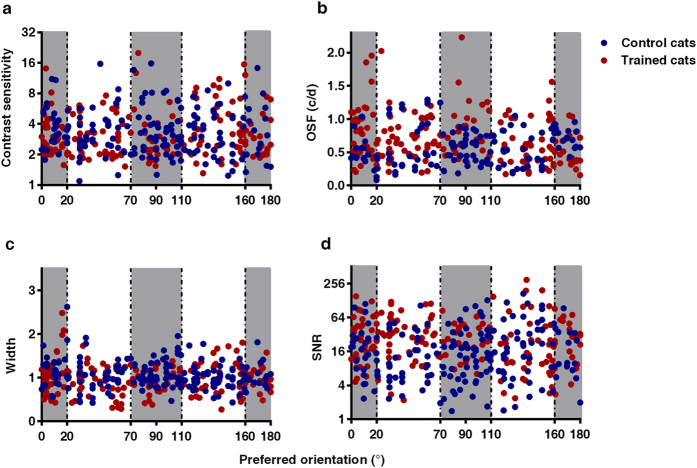
Effects of training on neuronal properties between neurons with different orientations. (**a–d**) represent distributions of contrast sensitivity, OSF, Width and SNR of neurons with preferred orientation near (±20°) (NN, gray background) and away (NA, white background) from the trained orientations for control (blue) and trained (red) cats. The learning effect was not specific to the trained orientation.

**Figure 5:**
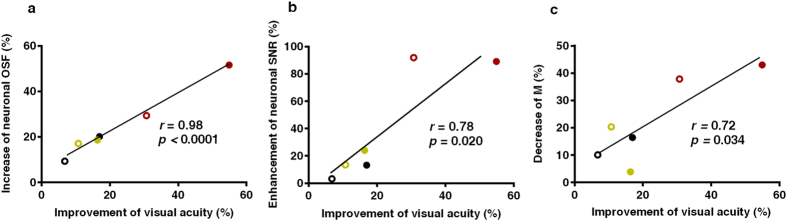
Correlation between the perceptual and net neuronal changes. The average neuronal responses of untrained cats were taken as baseline to calculate the ‘net’ neuronal changes. (**a**) Relationship between the improvements of visual acuities and the increase of average neuronal optimum spatial frequency values in the trained cats. (**b**) Relationship between the improvements of visual acuity and the enhancement of average neuronal SNR value in trained cats. (**c**) Relationship between the improvements of visual acuity and the decrease of average neuronal M value in trained cats. Each dot indicates one eye of a trained cat. Different color indicate different trained cat. Solid and empty circle represent trained and untrained eyes, respectively. Solid line in each subplot indicates the best linear fit.

The training apparatus was similar to that previously validated by Mitchell and colleagues^10^,^11^. The cats were trained to jump onto one of two adjacent platforms on which there were stimuli (jumping distance 50 cm). The adjacent two stimuli were displayed on one gamma-corrected Samsung monitor (SyncMaster P2450) that was held in a cradle so that its face pointed upward 7 cm beneath a glass plate, which was the surface onto which the cats were trained to jump (viewing distance 57 cm) (Fig. 1b). The mean luminance of stimuli was kept at 19 cd/m^2^, a luminance level that was matched to Hua *et al.*^5^. Since stimuli were displayed with a large field size (diameter 22°), eye fixation was not monitored.

Before the training, four cats received monocular conditioning practice in a two-alternative forced-choice sinusoidal grating orientation identification task with fixed, high-contrast (90%) grating stimuli at 0.3 or 0.4 c/d. Two side-by-side gratings, oriented horizontal and vertical respectively, were presented in each trial. Cats were trained to jump to the vertical grating. Correct answers were rewarded immediately with food and petting, whereas incorrect choices resulted in denial of these rewards. Trained cats concluded their conditioning training after high stable mean correct performance (>90%) was attained in about two months. This was then followed by pre-training assessment of contrast sensitivity functions (e.g. See Hua *et al.*^5^) and visual acuities in each eye. For visual acuity, the same grating orientation identification task was used. Percent accuracy at 0.6, 0.8, 1, 1.2, 1.5, 2, 2.4 and 3.0 c/d (50 trials for each SF, all intermixed) were measured to construct the frequency response curves. Visual acuity (VA) was defined as the spatial frequency corresponding to 75% accuracy. To obtain VA, we fit the visual acuity data with the equation below


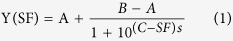


where *Y* is the accuracy rate. *SF* means the logarithm value of spatial frequency. *A*, *B* are the fitted smallest and biggest accuracy rates respectively. *C* is the logsf gives a correct rate half way between bottom and top and *s* is the slope of the curve.

After this, cats received monocular training of near-contrast threshold grating identification at a high spatial frequency (SF at which the contrast threshold was close to 0.5 in the pre-training assessment) for about 40 days with a randomly selected trained eye. The untrained eye was covered with a special mask that blocked light during training. A two-down/one-up staircase procedure was used to control the contrast in different trials, in which two consecutive correct responses resulted in a reduction of signal contrast (Cn + 1 = 0.9 Cn), and one wrong response resulted in an increase in contrast (Cn + 1 = 1.1 Cn), converging to a performance level of 70.7% correct^51^. In each daily training session, animals were given 250–300 trials in 5–6 blocks of 50 trials in each block. A 5–10 min break was provided between blocks. After the training stage, reassessment of post-training visual acuity was taken.

The magnitude of improvement for contrast sensitivity was calculated as:


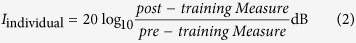


### Electrophysiological Recording and visual stimulation

Following the psychophysical experiment, three of the trained cats and four untrained cats were prepared for extracellular single unit recordings in area 17 (A17). The preparations were the same as that used in previous reports^5^,^52^ (For details, see Animal preparation in Supplementary). Tungsten microelectrodes (FHC), 1 × 32 linear Arrays (A1 × 32–6 mm–100–177, NeuroNexus) were used in these experiments. To access A17, we used vertical penetrations through craniotomies centered 4 mm posterior the interaural zero close to the midline.

Microelectrode signals as well as CRT timing data from a photocell (TSL12S, TAOS) were recorded using a data acquisition system (Cerebus, Blackrock Microsystems). All signals were broad-band filtered (0.3–7 kHz), digitized at 30 kHz, and saved for detailed offline data analysis.

Computer-controlled visual stimuli were displayed on a gamma-corrected CRT monitor (1024 × 768 pixels, 100 Hz, 19 cd/m^2^ mean luminance; Sony G220), placed at 57 cm from the animal. Custom software for stimulus generation and online data analysis was written in MATLAB (MathWorks) using Psychophysics Toolbox (PTB-3) extensions^53^,^54^.

All visual stimuli were presented monocularly. Pupils were maximally dilated with atropine (1%). Spectacle lenses and artifical pupil were used to make the eye clearly focused on the stimulus plane. The cell’s receptive field was carefully mapped by a series of computer-generated sinusoidal gratings presented on the monitor for each eye. To obtain the SF tuning properties of a neuron, we presented a series of sinusoidal gratings of different SFs (7 levels: 0.04, 0.08, 0.16, 0.32, 0.65, 1.30, 2.61 or 0.08, 0.16, 0.32, 0.65, 1.30, 2.61, 5.22 cycles/deg) with optimal size, preferred orientation and direction, fixed temporal frequency (3 Hz) and fixed high contrast (0.8). Each SF was presented 10 times in a randomized sequence. The contrast response properties of the neurons were obtained by randomly presenting a series of sinusoidal gratings of 9 different contrast levels (0.05, 0.07, 0.11, 0.15, 0.22, 0.33, 0.47, 0.69 and 1.0) in the same way of the SF tests. Each contrast was presented 15 or 20 times in a randomized sequence. A uniform mean luminance grey screen was displayed during all inter-trial intervals (pre 0.5 s and post 0.5 s for SF tests, pre 0.33 s and post 0.33 s for contrast tests) and also used as a “blank” condition (0.5 s for SF tests and 1.33 s for contrast tests) to measure spontaneous activities^55^.

### Data collection and analysis

The recorded Cerebus data files were first transformed to Plexon data file format using custom software. Single-unit isolation was achieved by manual spike waveform classification using Offline-Sorter software (Plexon).

For each cell, we recorded the responses to unilateral eye input for each eye, except the cells that had a strong monocular response (monocularity index larger than 0.75). Monocularity index (MI) was a folded version of the ocular dominance ratio^56^. Before full physiological recording, MI was estimated as follows:


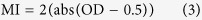


*OD* (ocular dominance ratio) was defined as (*I-M*)/((*I-M*) + (*C-M*)), where *I* was the response to ipsilateral eye, *C* was the response to contralateral eye, and *M* was the spontaneous firing level. The spontaneous activity (*M*) was acquired at the given blank stimuli.

The responses to SF of each neuron were subtracted by *M* and then fit with the logGaussian equation:


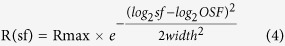


Where *Rmax* represents the neuron’s maximal visually evoked response to visual stimuli; *OSF* indicates the spatial frequency that evokes a neuron’s maximal response; *Width* is the standard deviation of the best fitting Gaussian function. A neuron’s SNR was defined as the ratio between the neuron’s visually evoked response to the optimal stimulus (*Rmax*) and the neuron’s spontaneous activity (*M*)^57^,^58^. Spontaneous activities below 1 spike/s were set equal to 1 spike/s for the signal-to-noise ratio analysis^57^. Cells with less than 85% goodness of fit were not included in our data analysis.

Statistical Analysis was carried out in SPSS (IBM, Chicago, IL, USA). The significance level was set at 0.05.

## Additional Information

**How to cite this article**: Ren, Z. *et al.* Neuronal basis of perceptual learning in striate cortex. *Sci. Rep.* **6**, 24769; doi: 10.1038/srep24769 (2016).

## Supplementary Material

Supplementary Information
