# Corrigendum: Neuronal basis of perceptual learning in striate cortex

**DOI:** 10.1038/srep41666

**Published:** 2017-02-10

**Authors:** Zhen Ren, Jiawei Zhou, Zhimo Yao, Zhengchun Wang, Nini Yuan, Guangwei Xu, Xuan Wang, Bing Zhang, Robert F. Hess, Yifeng Zhou

Scientific Reports 6: Article number: 24769; 10.1038/srep24769 published online: 04 20 2016; updated: 02 10 2017.

The Acknowledgements section in this Article is incomplete:

‘The authors thank Dr. Jiabo Tan, Mr. Shuya Han for valuable help in behavioral experiments. This work was supported by the National Natural Science Foundation of China (NSFC 81261120562 to YZ and NSFC 81500754 to JZ) and the Canadian Institutes of Health Research Grants (MOP-53346, CCI-125686 & MT-10818 to RFH). The funders had no role in study design, data collection and analysis, decision to publish, or preparation of the manuscript’.

Should read:

‘The authors thank Dr. Jiabo Tan, Mr. Shuya Han for valuable help in behavioral experiments. This work was supported by the National Natural Science Foundation of China (NSFC 31571074 and NSFC 81261120562 to YZ and NSFC 81500754 to JZ) and the Canadian Institutes of Health Research Grants (MOP-53346, CCI-125686 & MT-10818 to RFH).The funders had no role in study design, data collection and analysis, decision to publish, or preparation of the manuscript’.

