# Supplementary material for: Neuronal basis of perceptual learning in striate cortex
Source: Sci Rep. 2016 Apr 20;6:24769. doi: 10.1038/srep24769 (PMC4837366; doi:10.1038/srep24769)
Supplement: Supplementary Information [file srep24769-s1.pdf]

Supplementary Online Material for

Neuronal basis of perceptual learning in striate cortex

Zhen Ren[1], Jiawei Zhou[2,3*], Zhimo Yao[1], Zhengchun Wang[1], Nini Yuan[1], Guangwei Xu[1], Xuan Wang[1], Bing Zhang[1], Robert F. Hess[3] and Yifeng Zhou[1*]

1. CAS Key Laboratory of Brain Function and Disease, and School of Life Sciences, University of Science and Technology of China, Hefei, Anhui, PR China,

2. School of Ophthalmology and Optometry and Eye hospital, Wenzhou Medical University, Wenzhou, Zhejiang 325003, PR China,

3. McGill Vision Research, Department of Ophthalmology, McGill University, Montreal, Quebec, Canada.

**Correspondence and requests for materials should be addressed to: jiawei.zhou@mcgill.ca (J.Z.) and zhouy@ustc.edu.cn (Y.Z.).*

Table S1. Differences of measurements of grating acuity between the current and previous studies

	Mitchell et al, 2003 Duffy & Mitchell, 2013	The current study	Expected direction of effect
Stimuli	Sharp-edged Square Wave grating	Edge-blurred Sinewave grating	++
Contrast	100% contrast (Printed paper)	90% contrast (Gamma-corrected LED screen)	+
Procedure	Block design: low to high SFs with small step size	Randomized Design: SFs in different trials with larger step size	+++++
Threshold Accuracy	70%	75%	+
Luminance	55 cd/m^2	19 cd/m^2	+++
Viewing distance	72 cm (a head movement of 5 cm could induce a 7% variance of SF)	57 cm (a head movement of 5 cm could induce a 9% variance of SF)	0

Behavioral spatial frequency response curves

Before the training, visual acuity curves were not significantly related to eye (F (1, 48) = 0.253, *p* = 0.617; Figure S1). After training, the visual acuity curves of trained cats were significantly elevated (F (1, 96) = 26.587, *p* < 0.001; Figure S1).

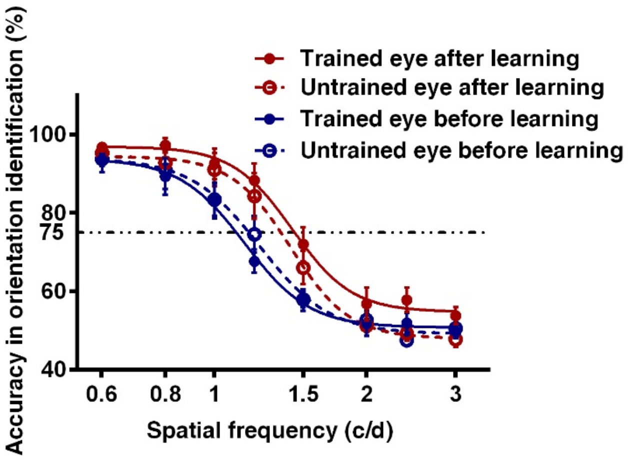

Figure S1. The average visual acuity curves of the four trained cats. Blue symbols

and lines represent results before the training, red symbols and lines represent results after the training, solid symbols and lines represent results of the trained eye and dashed symbols and lines represent results of the untrained eye. Each dot indicates the measured accuracy at each spatial frequency, curves indicate the fitted data. The black dashed horizontal lines mark the 75% accuracy levels, which indicates the visual acuity in terms of spatial frequency.

Changes of simple and complex cells in A17

The proportions of simple and complex cells were similar in control and trained groups (Fisher's exact test, $p = 0.54$). For simple cells, the contrast sensitivity (t (47) = 0.023, p= 0.982) and tuning width (t (47) = 1.431, p= 0.159) between the two groups were not significantly different, but the trained group exhibited significantly higher OSF (Mann-Whitney U test, $p = 0.011$) and greater SNR (t (47) = 2.041, p= 0.047) than the control group did. For complex cells, like that of simple cells there were neither a significant difference in contrast sensitivity (t (322) = 0.796, p= 0.426) nor tuning width (t (322) = 1.900, p= 0.058) between the two groups, but a significantly higher OSF (Mann-Whitney U test, $p = 0.001$) and greater SNR (Mann-Whitney U test, $p < 0.001$) in the trained cats.

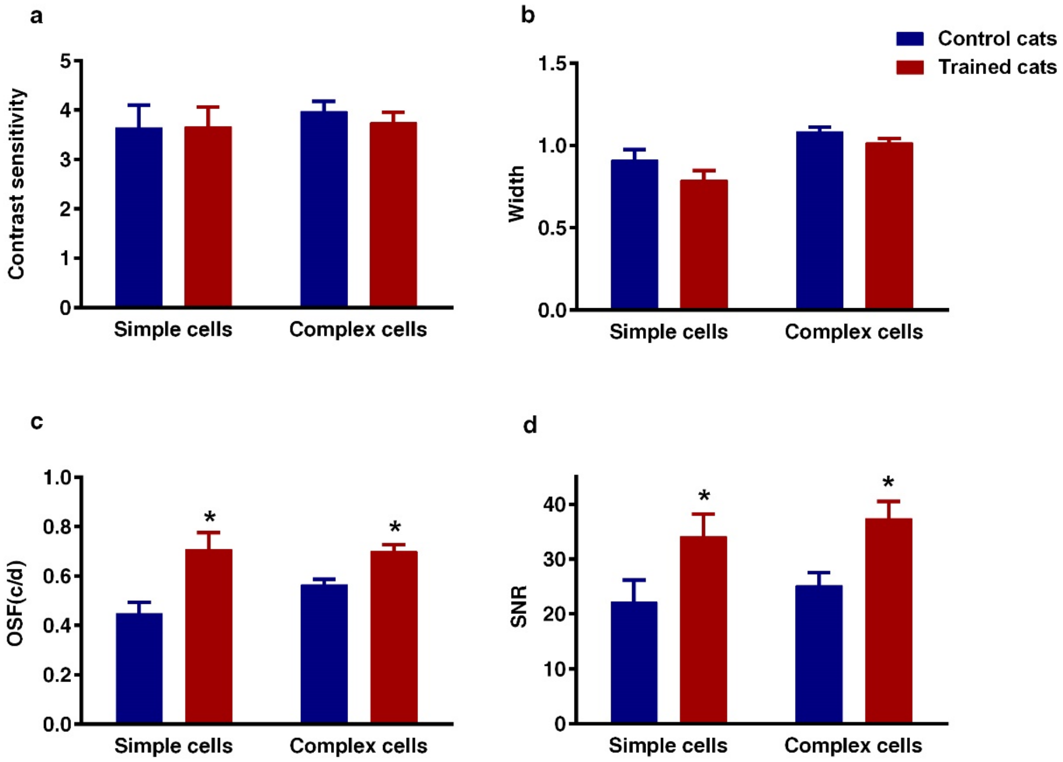

Figure S2. Comparison of Contrast sensitivity (a), Width (b), OSF (c) and SNR (d) of simple and complex cells between control (blue) and trained (red) cats. Simple and complex cells exhibited similar trained-related changes: increased neuronal OSF (c) and SNR (d). "*" indicated $p < 0.05$.

Changes of Rmax and M for cells in A17

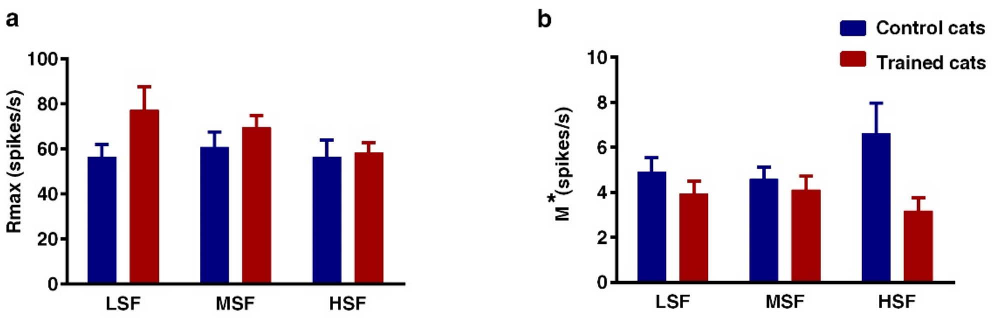

Figure S3. Comparison of Rmax (a) and M (b) for V1 neurons in control (blue) and trained (red) cats. LSF, MSF and HSF represent neurons with low spatial frequency (<0.45 cpd), medium spatial frequency (0.45-0.85 cpd) and high spatial frequency (>0.85 cpd) responses, respectively. Error bars indicate SEM.

a. There were no significant training effects on Rmax (F (1,367) = 3.158, p = 0.076).

b. Training significantly decreased the M (spontaneous activities) of V1 neurons (F (1,367) = 8.225, p = 0.004).

Method

Animal preparation

Seven healthy adult cats (weights between 2.5kg and 3.5kg) underwent electrophysiological recordings. Corrected spectacle lenses were used as needed during recording. All procedures were approved by the Animal Care and Use Committee of University of Science and Technology of China and in accordance with the National Institutes of Health Guide for the Care and Use of Laboratory Animals.

Anesthesia was induced with ketamine HCL (20 mg/kg, *i.m.*). After the intravenous and tracheal cannulae were inserted, cats were placed in a stereotaxic apparatus with ear bars, and bite bar. A long-acting anaesthetic (2% lidocaine-HCl jelly) and Marcaine (0.25%) were applied to all pressure points and wound margins, respectively. Neosynephrine (5%) and atropine sulfate (1%) were topically administered to retract nictitating membranes and dilate pupils, respectively. A pair of zero-power air-permeable contact lenses was fitted to protect the corneas. Lactated Ringer's solution (LRS) with 2.5% dextrose was given through a venous cannula (5-10 ml/kg/h). Gallamine triethiodide was delivered in the LRS solution (10 mg/kg/h) to maintain paralysis. Propofol (5mg/kg/h, *i.v*) and sufentanil (10ng/kg/h, *i.v*) were used to maintain anesthesia during the whole recording process. Rectal temperature was maintained around 38℃ with a heating blanket. Expired CO_2 was maintained between 3.8% and 4.3%. The heart rate, electrocardiogram, and cortical electrical activity were monitored throughout the experiment to assess the level of anesthesia. Penicillin (200000U, *i.m.*), dexamethasone (5 mg, *i.m.*) and atropine (1 mg, *i.m.*) were administered every 12 h throughout the experiment.

To measure contrast sensitivity of V1 neurons

We used the same method as Anzail et al.'s study (*Contrast coding by cells in the cat's striate cortex: Monocular vs. binocular detection*). First, for every stimuli, each cycle was regarded as a trial and we obtained spike frequency histograms (SFHs) (Figure S4b). Then the probability that a spike rate drawn from a spike frequency distribution of responses to a stimulus exceeds a criterion spike rate was defined as a hit probability. The probability that a spike rate drawn from a spike frequency

distribution of spontaneous activity exceeds the criterion spike rate was defined as a false probability (Figure S4b). By varying the criterion spike rate from zero to infinitely large, pairs of hit and false alarm probabilities were obtained to generate an ROC curve. The areas under ROC curves represent the response probabilities which take values from 0.5 to 1 (Figure S4c). At last, Response probabilities plotted against stimulus contrast formed an S-shaped curve which was fitted by the cumulative Weibull function described by equation (1), and the contrast which gave a response probability of 0.75 was defined as contrast threshold (Figure S4d).

$$P(x) = s- (s-c) \exp [-(x/a)^{b}] \qquad (1)$$

Where x and P are contrast and response probability, respectively. Parameters a, b, c and s represent horizontal position, steepness, chance probability, and saturation probability of the function, respectively. The parameter c is fixed at 0.5.

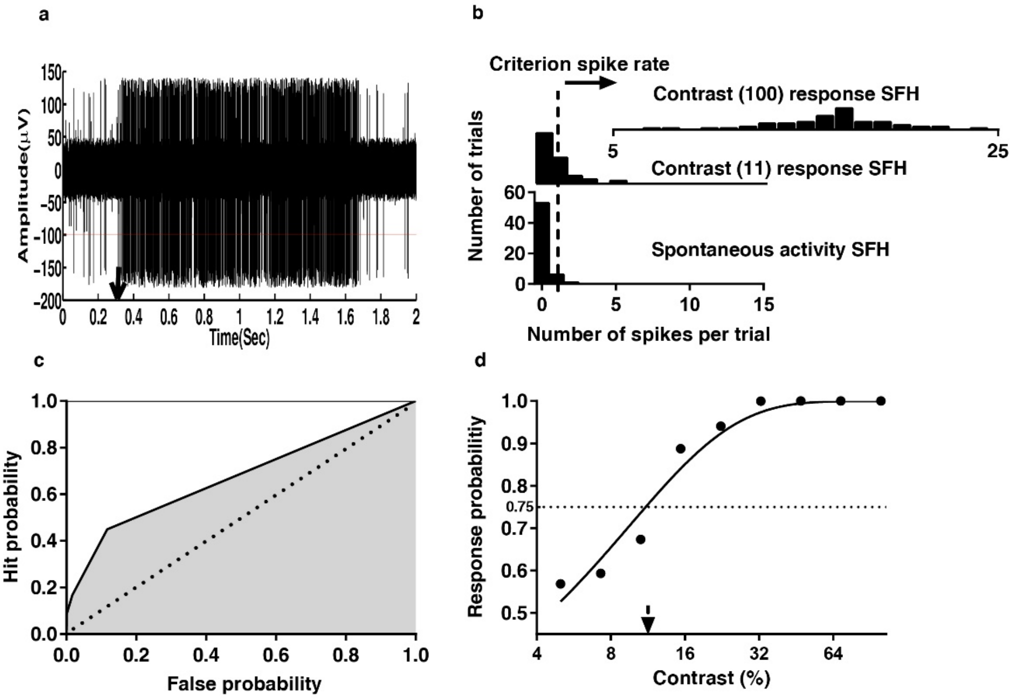

Figure S4. Examples of neuronal response to contrast. a. The voltage trace of a neuron's response to its optimal stimulus (SF= 0.82 c/d, contrast = 100%). A spike with amplitude surpassing the horizontal red line is counted as an action potential. The neuron's response is evoked by 4 cycles of grating stimulation (1.33s), and the spontaneous activity is acquired by a blank stimulus with the same duration. The

arrow indicates the stimulus onset time.

b. An example of neuronal spike frequency histograms (SFHs) (bottom: spontaneous activity, middle: contrast = 11%, top: contrast = 100%). For a criterion spike rate, a hit probability and a false probability are defined as the right areas of the vertical dashed line divided by the total area for the contrast response SFH and for the spontaneous activity SFH, respectively. The criterion spike rate is changed from 0 to infinitely large to obtain pairs of hit and false alarm probabilities for a given stimulus contrast.

c. ROC Curve. When a contrast response spike frequency histogram (SFH) overlaps completely with a spontaneous activity SFH, its ROC curve runs along the diagonal and the response probability (50%) is shown as area between the dashed line and x axis. When the contrast increases, for example to 11%, the ROC curve becomes arched and the response probability increases as shown by the gray area.

d. Response probabilities vs. contrast. The contrast which gave a response probability of 0.75 was defined as contrast threshold, as shown with arrow.
